# Ectopic expression of C-terminal tubulin variants alters wood composition and structure in *Populus*

**DOI:** 10.1186/1753-6561-5-S7-P76

**Published:** 2011-09-13

**Authors:** Prashant Swamy, Feng Long, Batbayar Nyamdari, Jeng-Der Chung, Shawn Mansfield, Scott Harding, Chung-Jui Tsai

**Affiliations:** 1 University of Georgia, USA; 2 Taiwan Forestry Research Institute, Taiwan; 3 University of British Columbia, Canada

## 

Cortical microtubules are cytoskeletal components that are relevant to the bioenergy and forest products industry due to their postulated role in orchestrating cellulose microfibril deposition during cell wall formation. The microtubule component proteins α- (TUA) and β-tubulins (TUB) are encoded by multi-gene families with very high overall sequence homology across species. To advance our initial characterization studies (Oakley et al., 2007), we have developed a suite of transgenic *Populus* that exhibit perturbed *TUA* to *TUB* transcript ratios, or that express tubulin PTM mimics. Most of the construct combinations resulted in abnormal organogenesis and vascular development, and failed to produce viable plants. Only three of the combinations led to whole-plant regeneration, and interestingly, all three featured the C-terminal variants. This is significant because the C-terminus is the post-translational modification (PTM) hot-spot in animal tubulins. One of the PTM mimics that we developed lacks a C-terminal tyrosine, thought to be the target of a tyrosine cleavage and re-ligation cycle important for tubulin regulation in animal cells. The transgenic trees appeared morphologically normal, but exhibited a range of epinasty and twisting phenotypes in mature leaves. Bark color was noticeably lighter in the transgenics. Microfibril angle, wood density, lignin content, lignin structure and metabolic profiles were altered in the transgenic wood. The results are consistent with a function of microtubules and microtubule PTMs for plant development and cell wall biogenesis in *Populus*, and offer novel strategies to manipulation of wood properties.

